# DNA copy number changes in high-grade malignant peripheral nerve sheath tumors by array CGH

**DOI:** 10.1186/1476-4598-7-48

**Published:** 2008-06-03

**Authors:** Stine H Kresse, Magne Skårn, Hege O Ohnstad, Heidi M Namløs, Bodil Bjerkehagen, Ola Myklebost, Leonardo A Meza-Zepeda

**Affiliations:** 1 Department of Tumor Biology, Radiumhospitalet, Rikshospitalet, Oslo, Norway; 2 Faculty of Medicine, University of Oslo, Norway; 3 Pathology Clinic, Radiumhospitalet, Rikshospitalet, Oslo, Norway; 4 Norwegian Microarray Consortium, Department of Molecular Biosciences, University of Oslo, Norway

## Abstract

**Background:**

Malignant peripheral nerve sheath tumors (MPNSTs) are rare and highly aggressive soft tissue tumors showing complex chromosomal aberrations. In order to identify recurrent chromosomal regions of gain and loss, and thereby novel gene targets of potential importance for MPNST development and/or progression, we have analyzed DNA copy number changes in seven high-grade MPNSTs using microarray-based comparative genomic hybridization (array CGH).

**Results:**

Considerable more gains than losses were observed, and the most frequent minimal recurrent regions of gain included 1q24.1-q24.2, 1q24.3-q25.1, 8p23.1-p12, 9q34.11-q34.13 and 17q23.2-q25.3, all gained in five of seven samples. The 17q23.2-q25.3 region was gained in all five patients with poor outcome and not in the two patients with disease-free survival. cDNA microarray analysis and quantitative real-time reverse transcription PCR were used to investigate expression of genes located within these regions. The gene lysyl oxidase-like 2 (*LOXL2*) was identified as a candidate target for the 8p23.1-p12 gain. Within 17q, the genes topoisomerase II-α (*TOP2A*), ets variant gene 4 (E1A enhancer binding protein, *E1AF*) (*ETV4*) and baculoviral IAP repeat-containing 5 (survivin) (*BIRC5*) showed increased expression in all samples compared to two benign tumors. Increased expression of these genes has previously been associated with poor survival in other malignancies, and for *TOP2A*, in MPNSTs as well. In addition, we have analyzed the expression of five micro RNAs located within the 17q23.2-q25.3 region, but none of them showed high expression levels compared to the benign tumors.

**Conclusion:**

Our study shows the potential of using DNA copy number changes obtained by array CGH to predict the prognosis of MPNST patients. Although no clear correlations between the expression level and patient outcome were observed, the genes *TOP2A*, *ETV4 *and *BIRC5 *are interesting candidate targets for the 17q gain associated with poor survival.

## Background

Malignant peripheral nerve sheath tumors (MPNSTs) are rare tumors that arise sporadically or as part of the neurofibromatosis type 1 (NF1) or -2 (NF2) autosomal inherited disorder. The NF1/von Recklinghausen neurofibromatosis, caused by germ line mutations of the *NF1 *tumor suppressor gene, is one of the most common autosomal dominant inherited disorders, occurring at a frequency of one in every 4,000 individuals [1]. Patients with this disease have an increased risk of benign and malignant tumors [2]. In contrast to other soft tissue malignancies, the majority of MPNSTs derives from previously existing neurofibromas [3].

Cytogenetically, MPNSTs have complex karyotypes with multiple losses frequently observed in chromosome regions 1p, 9p, 17q and 22 [4,5]. Several comparative genomic hybridization (CGH) studies have revealed a higher frequency of gains compared to losses, involving chromosome regions 5p, 7p, 7q, 8q and 17q [6-10]. Recurrent gain of 7p15-p21 and 17q22-qter has been associated with poor overall survival [10], and increased copy number and expression of topoisomerase II-α (*TOP2A*) in 17q21.2 have been associated with poor cancer-specific survival and presence of metastasis [11].

In order to identify specific genomic events and candidate targets that may play a role in MPNST development and/or progression, we have used microarray-based CGH (array CGH) to map the distribution and frequency of DNA copy number changes in seven high-grade MPNSTs. cDNA microarray analysis and quantitative real-time reverse transcription PCR (RT-PCR) were used to investigate expression of genes located within the most frequently altered chromosomal regions. In addition, micro RNA (miRNA) expression in a recurrent region of gain was determined using quantitative real-time RT-PCR.

## Results

### Recurrently altered chromosomal regions in MPNSTs

**Figure 1:**
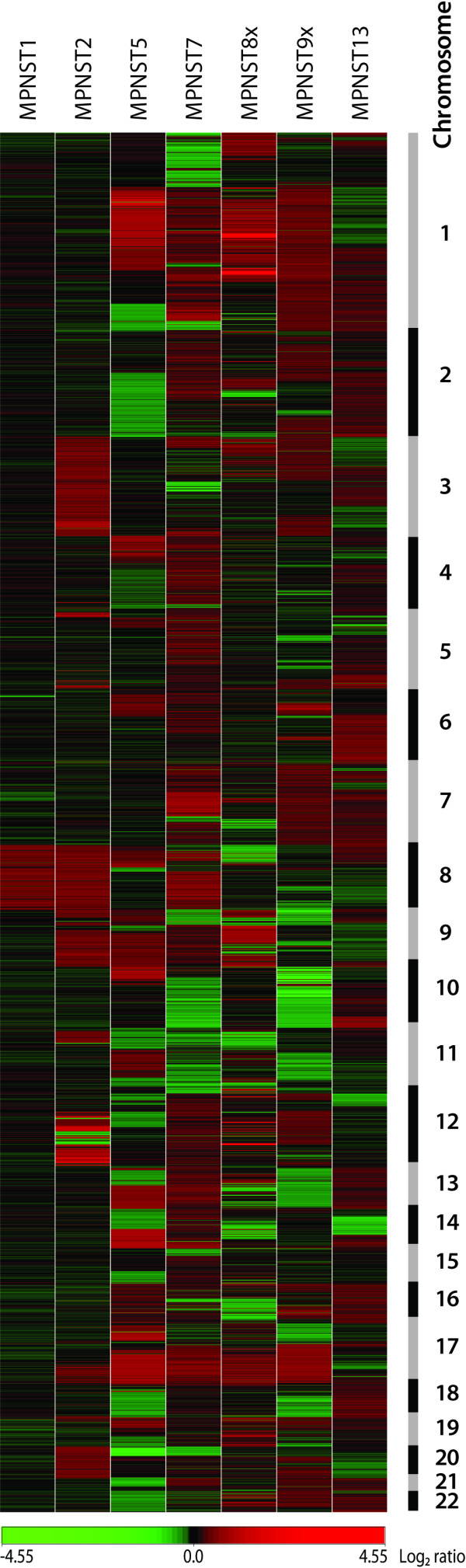
Heat map of DNA copy number ratios of seven MPNSTs relative to a pool of normal diploid DNA. A total of 3,167 unique genomic clones are shown in chromosomal order from 1ptel to 22qtel. Chromosomes are indicated with black and grey bars. Red, increases in DNA copy number; green, decreases in DNA copy number.

DNA copy number changes in seven high-grade MPNSTs (Table 1) were analyzed using a 1 Mb resolution bacterial- and P1 artificial chromosome (BAC and PAC) genomic microarray supplemented with the tiling-path between 1q12 and the beginning of 1q25. A heat map of DNA copy number ratios of the tumor samples is shown in Figure 1.

**Table 1:** Clinical data for tumor samples

Sample	Sample origin	Patient age (years)/sex	Diagnosis Initial	Revised	Grade^1^	Location	Size (cm)^2^	Metastasis (months)^3^	Status	Follow-up (months)^4^	Neurofibromatosis
MS1	Prim	71/F	MPNST	MPNST	4	Upper trunk	10	NM	NED	100	+
MS2	Prim	46/M	MPNST	MPNST	4	Lower leg	40	MD	DD	1	+
MS5	Met	24/M	MPNST	MPNST	4	Upper trunk	9	20	DD	163	
MS7	Prim	42/M	MPNST	MPNST	4	Pelvic areas	7	6	DD	12	
MS8x	Prim	78/F	MPNST	MPNST	4	Gluteal	20	MD	DD	12	
MS9x	Rec	26/M	MPNST	MPNST	4	Upper trunk	4	70	DD	79	+
MS13	Prim	40/M	MFH	MPNST^5^	4	Thigh	10	NM	NED	216	+
BS	Prim	50/M	MPNST	BS	-	Retroperitoneum	7	NM	NED	120	
NF	Prim	19/F	MPNST	NF	-	Retroperitoneum	11	185	DD	186	

Abbreviations: x, xenograft; Prim, primary tumor; Met, metastasis; Rec, recurrence; F, female; M, male; MFH, malignant fibrous histiocytoma; BS, benign schwannoma; NF, neurofibroma; NM, no metastasis; MD, metastasis at diagnosis; NED, no evidence of disease; DD, dead of disease.

^1 ^Grading is based on a four-tiered system used in the Scandinavian Sarcoma Group.

^2 ^Largest diameter of the tumor.

^3 ^Time to first metastasis from diagnosis.

^4 ^Time to last follow-up from diagnosis.

^5 ^Pleomorphic sarcoma in a patient with neurofibromatosis.

**Figure 2:**
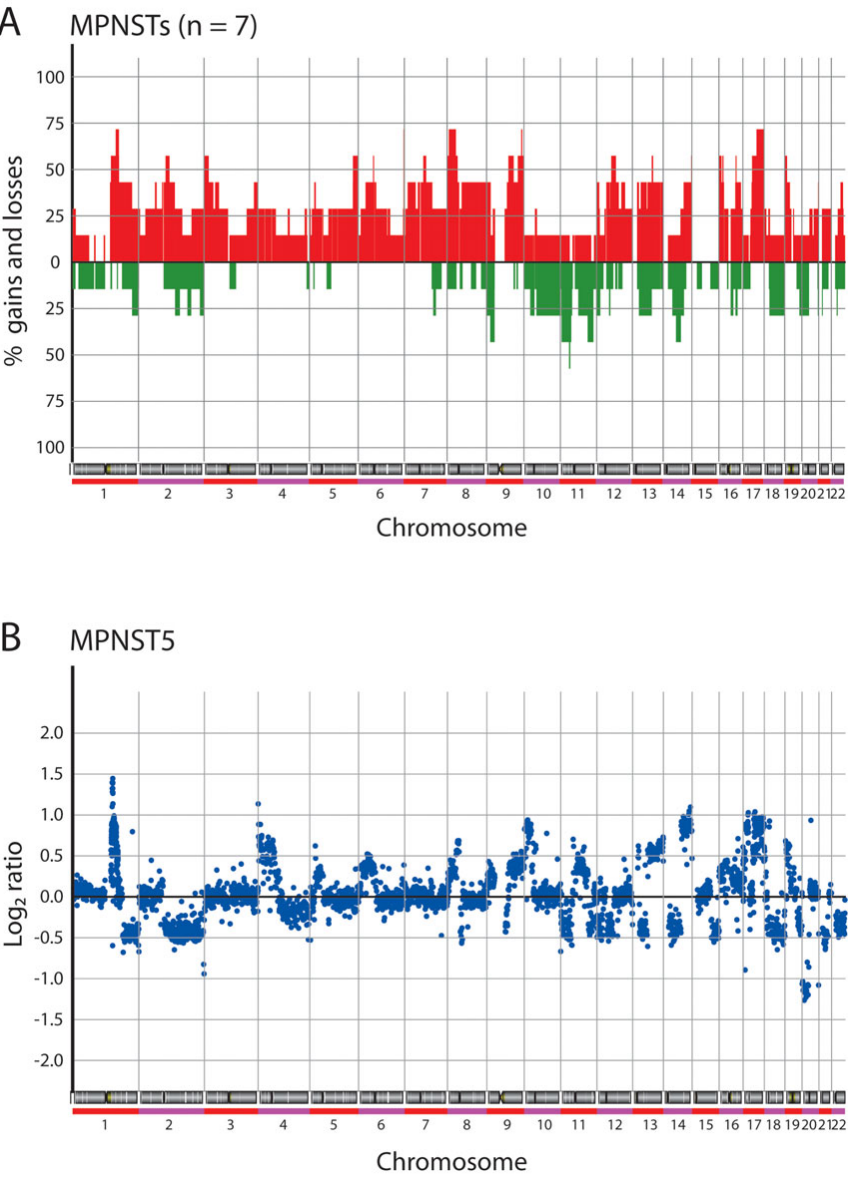
**(A) **Genome-wide frequency plot of copy number alterations identified by ACE in seven MPNSTs. Red, increases in DNA copy number; green, decreases in DNA copy number. **(B) **Representative whole genome DNA copy number profile for MPNSTs. Log_2 _ratio for each of the genomic clones is plotted according to chromosome position.

Regions with significant DNA copy number changes in each sample were identified using the "Analysis of Copy Errors" (ACE) algorithm in CGH-Explorer. The resulting frequency plot of gains and losses is shown in Figure 2a, and a representative ratio plot for this type of tumors in Figure 2b. Genome-wide ratio plots for all samples are shown in Additional file 1. Minimal recurrent regions of alteration identified by ACE in at least three of seven (≥ 43%) samples are presented in Table 2. The complete list of data of all defined regions of gain and loss from the ACE analysis is presented in Additional file 2.

**Table 2:** Minimal recurrent regions altered in MPNSTs (n = 7)

Cytoband	Aberration	Start clone	End clone	Size (Mb)	Frequency	Observation
1q21.1	Gain	RP3-365I19	RP11-544O24	1.2	3/7	1 sample with +1q
1q24.1-q24.2	Gain	RP11-525G13	RP4-780M13	3.7	5/7	1 sample with +1q
1q24.3-q25.1	Gain	RP1-127D3	RP5-830A10	3.5	5/7	1 sample with +1q
2p15-p14	Gain	RP11-52F10	RP11-263L17	3.0	3/7	1 sample with +2p
2q11.2-q13	Gain	RP11-451C2	RP11-368A17	11.0	4/7	1 sample with +2q
3p26.2-p25.1	Gain	RP11-95E11	RP11-255O19	12.6	4/7	1 sample with +3, 1 sample with +3p
3p22.1-p21.1	Gain	RP11-437N10	RP11-122D19	11.5	3/7	1 sample with +3, 1 sample with +3p
3q27.2-q29	Gain	RP11-110C15	RP11-23M2	12.5	3/7	1 sample with +3
5p14.3	Gain	RP11-28P24	RP11-374E21	2.0	3/7	1 sample with +5
5q34-q35.3	Gain	CTC-320C6	RP11-281O15	15.2	4/7	1 sample with +5
6p22.1-p12.1	Gain	RP11-373N24	RP11-472M19	28.1	3/7	1 sample with +6p
6p12.1-q12	Gain	RP3-422B11	RP11-349P19	7.8	3/7	1 sample with +6p, 1 sample with +6q
7p21.2-p14.1	Gain	RP11-512E16	RP5-1178G13	26.3	3/7	1 sample with +7
7q11.23-q21.11	Gain	RP11-313P13	RP5-1057M1	7.4	4/7	1 sample with +7, 1 sample with +7q
8p23.1-p12	Gain	RP11-540E4	RP11-473A17	22.9	5/7	2 samples with +8
8q11.23-q24.3	Gain	RP11-53M11	RP5-1056B24	90.5	3/7	2 samples with +8
9p24.3-p23	Gain	RP11-48M17	RP11-413D24	11.6	3/7	1 sample with +9p
9p22.3-p21.2	Loss	RP11-490C5	RP11-20P5	12.8	3/7	2 samples with -9p
9q21.32-q22.33	Gain	RP11-541F16	RP11-192E23	16.0	4/7	
9q34.11-q34.13	Gain	RP11-202H3	RP11-143H20	1.0	5/7	
11p13	Loss	RP1-316D7	RP4-607I7	1.5	4/7	
11q22.3-q23.1	Loss	RP11-2I22	RP11-11N15	7.7	3/7	
11q23.2-q23.3	Loss	RP11-212D19	RP11-142I2	7.0	3/7	
12p13.32-p13.31	Gain	RP11-543P15	RP11-277E18	4.8	3/7	1 sample with +12
12q12-q13.11	Gain	RP11-333D23	RP11-89H19	9.3	3/7	1 sample with +12
12q13.3-q15	Gain	RP11-474N8	RP11-101K2	14.2	4/7	1 sample with +12
12q21.2-q21.31	Gain	RP11-26L7	RP11-268A19	3.3	3/7	1 sample with +12
12q22	Gain	RP11-24I19	RP11-372G13	0.8	3/7	1 sample with +12
12q22-q23.3	Gain	RP11-410A13	RP11-415D21	9.1	3/7	1 sample with +12
13q12.11-q12.12	Gain	RP11-76K19	RP11-760M1	3.9	3/7	1 sample with +13
13q13.1-q14.3	Gain	RP11-141M1	RP11-327P2	18.5	3/7	1 sample with +13
13q22.1-q22.2	Gain	RP11-552M6	RP11-332E3	2.1	4/7	1 sample with +13
14q21.3-q23.3	Loss	RP11-346L24	RP11-430G13	15.3	3/7	
14q24.3-q32.33	Gain	RP11-61F4	RP11-417P24	27.9	3/7	1 sample with +14
16p13.3-p13.2	Gain	RP11-344L6	RP11-148F10	7.9	4/7	2 samples with +16
16p13.12-p13.11	Gain	RP11-82O18	RP11-489O1	0.9	4/7	2 samples with +16
16q21-q23.2	Gain	RP11-148F12	RP11-437L22	18.2	3/7	2 samples with +16
17q23.2-q25.3	Gain	RP11-112J9	RP11-567O16	26.2	5/7	
19p13.3-p13.2	Gain	CTB-31C16	RP11-492L14	4.9	4/7	2 samples with +19p
22q12.3-q13.2	Gain	LL22NC01-132D12	RP3-437M21	6.1	3/7	

The tumors showed considerably more recurrent gains than losses. Thirty-five of the 40 identified recurrent regions of alteration were gains, compared with only five regions of loss. The most frequent regions of increased copy number were in 1q, 8p, 9q and 17q, all detected in five of seven tumors. In 1q, three minimal recurrent regions of gain were identified; 1q24.1-q24.2 (3.7 Mb) and 1q24.3-q25.1 (3.5 Mb) in five of seven tumors and 1q21.1 (1.2 Mb) in three tumors. The other regions gained in five tumors were 8p23.1-p12 (22.9 Mb), 9q34.11-q34.13 (1.0 Mb) and 17q23.2-q25.3 (26.2 Mb). Ten minimal recurrent regions of gain were observed in four tumors; 2q11.2-q13 (11.0 Mb), 3p26.2-p25.1 (12.6 Mb), 5q34-q35.3 (15.2 Mb), 7q11.23-q21.11 (7.4 Mb), 9q21.32-q22.33 (16 Mb), 12q13.3-q15 (14.2 Mb), 13q22.1-q22.2 (2.1 Mb), 16p13.3-p13.2 (7.9 Mb), 16p13.12-p13.11 (0.9 Mb) and 19p13.3-p13.2 (4.9 Mb). In addition, 20 regions of gain were identified in three tumors (see Table 2). High-level amplification (log_2 _ratio > 1) was observed in some of the tumors, mainly of regions in 1q and 12q [see Additional file 2].

Three of five identified minimal recurrent regions of loss were located in chromosome 11. Four tumors showed loss of 11p13 (1.5 Mb), whereas 11q22.3-q23.1 (7.7 Mb) and 11q23.2-q23.3 (7.0 Mb) were lost in three tumors. In addition, loss of 9p22.3-p21.2 (12.8 Mb) and 14q21.3-q23.3 (15.3 Mb) were observed in three tumors. Homozygous deletion (log_2 _ratio < -1) was observed in some of the tumors, mainly of regions in 9p [see Additional file 2].

### Gene expression in frequently altered chromosomal regions

Gene expression has previously been analyzed using cDNA microarrays in a panel of soft tissue sarcomas, including six of the MPNSTs studied here (using the xenograft of MPNST2) [12]. In order to identify candidate target genes for the DNA copy number changes, the expression level of genes located within the most frequently altered regions (gained in five of seven samples) was investigated. Genes with increased expression relative to the median for soft tissue sarcomas (log_2 _ratio > 1) in two or more of the six MPNSTs analyzed were identified.

Within the minimal recurrent region of gain in 8p23.1-p12, six genes showed increased expression [12]. Two genes were over-expressed in three samples; lysyl oxidase-like 2 (*LOXL2*) and zinc finger protein 395 (*ZNF395*). In two of the samples, the genes mitochondrial tumor suppressor 1 (*MTUS1*), leucine zipper, putative tumor suppressor 1 (*LZTS1*), scavenger receptor class A, member 3 (*SCARA3*) and UBX domain-containing protein 6 (Reproduction 8 protein) (Protein Rep-8) (*UBXD6*) were over-expressed (data not shown).

**Figure 3:**
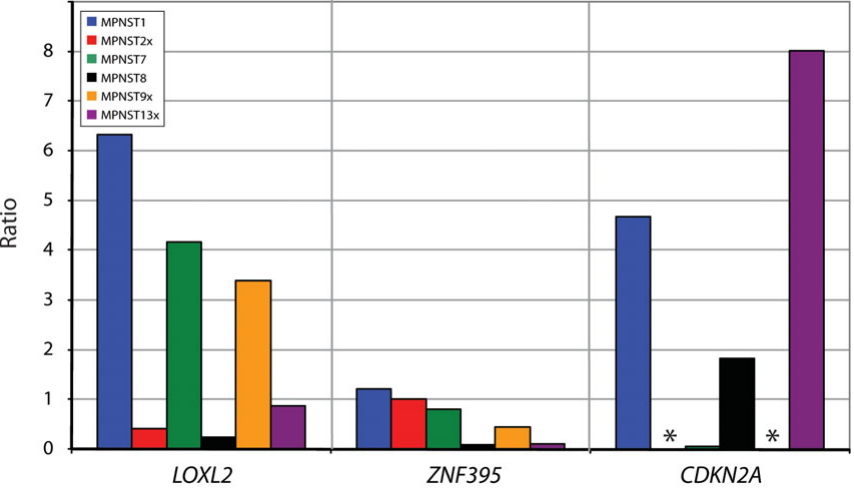
Plot of expression level of three candidate target genes in six of the MPNSTs, normalized with the average expression of three endogenous controls (*B2M*, *GAPDH *and *TBP*). The expression level was determined relative to the average expression of two benign tumors. No detection of PCR-product is indicated with an asterisk (*).

The expression level of *LOXL2 *and *ZNF395 *was in addition determined using quantitative real-time RT-PCR in six of the samples (using the xenograft of MPNST2 and -13 and the patient sample of MPNST8x). The expression level was compared to the average expression in two benign tumors, one benign schwannoma (BS) and one neurofibroma (NF) (Table 1). Figure 3 shows the relative expression level of *LOXL2 *and *ZNF395*. *LOXL2 *showed increased expression in MPNST1, -7 and -9x, whereas *ZNF395 *showed approximately similar expression levels in the MPNSTs and the benign tumors.

In the minimal recurrent regions of gain in 1q24.1-q24.2, 1q24.3-q25.1 and 9q34.11-q34.13, no genes present in the cDNA microarray showed increased expression in more than one of the samples.

Homozygous deletion (log_2 _ratio < -1) of the region containing the tumor suppressor gene cyclin-dependent kinase inhibitor 2A (melanoma, p16, inhibits CDK4) (*CDKN2A*) was observed in two samples, and heterozygous deletion in one sample [see Additional file 2]. The expression level of *CDKN2A *was determined using quantitative real-time RT-PCR in six of the samples (Figure 3). The three samples with deletion of the region, MPNST2x, -7 and -9x, showed no or low expression compared to the benign tumors, whereas the other three MPNSTs showed increased expression.

### Characterization of the 17q gain

**Figure 4:**
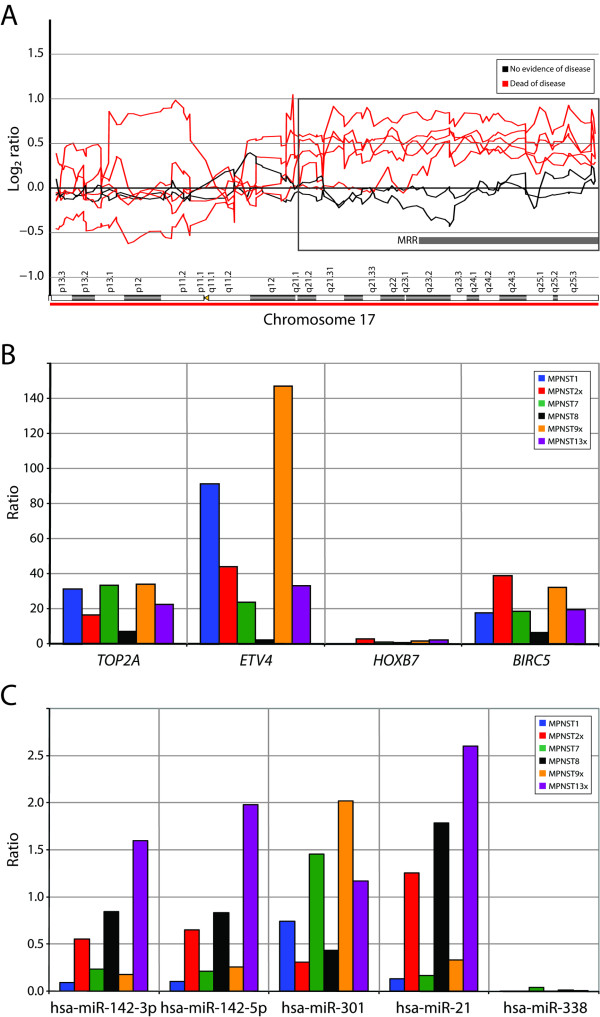
**(A) **DNA copy number profile of chromosome 17 for seven MPNSTs. Log_2 _ratio for each of the genomic clones is plotted according to chromosome position using "moving average smoothing" with a three-clone window. The minimal recurrent region (MRR) gained in all five patients with poor outcome is indicated, as well as the region where at least four of the five patients showed gain (highlighted by a square). **(B) **Plot of expression level of four candidate target genes in 17q in six of the MPNSTs, normalized with the average expression of three endogenous controls (*B2M*, *GAPDH *and *TBP*). The expression level was determined relative to the average expression of two benign tumors. **(C) **Plot of expression level of five candidate target miRNAs in 17q in six of the MPNSTs, normalized with the average expression of two endogenous controls (RNU6B and -24). The expression level was determined relative to the average expression of two benign tumors.

The minimal recurrent region 17q23.2-q25.3 was gained in all patients who died of the disease (five of seven patients), but not in the two patients with disease-free survival (MPNST1 and -13, with follow-up of 100 and 216 months, respectively). Figure 4a shows the copy number of chromosome 17 for all the tumor samples.

The expression level of genes located within the minimal recurrent region, as well as the proximal region where only four of seven samples showed gain (17q21.2-q23.2), was examined. By cDNA microarray analysis, ten genes showed increased expression relative to the median for soft tissue sarcomas (log_2 _ratio > 1) in two or more of the six MPNSTs analyzed [12]. Three genes were over-expressed in three MPNSTs; baculoviral IAP repeat-containing 5 (survivin) (*BIRC5*), ets variant gene 4 (E1A enhancer binding protein, *E1AF*) (*ETV4*) and homeobox B7 (*HOXB7*). In two of the samples, the genes RAB5C, member RAS oncogene family (*RAB5C*), vesicle amine transport protein 1 homolog (T. californica) (*VAT1*), distal-less homeobox 4 (*DLX4*), DEAD (Asp-Glu-Ala-Asp) box polypeptide 5 (*DDX5*), thymidine kinase 1, soluble (*TK1*), stimulated by retinoic acid 13 homolog (mouse) (*STRA13*) and solute carrier family 16, member 3 (monocarboxylic acid transporter 4) (*SLC16A3*) were over-expressed (data not shown).

The expression level of *ETV4*, *HOXB7 *and *BIRC5 *was in addition determined using quantitative real-time RT-PCR in six of the samples. The expression level of *TOP2A *was also determined, since increased expression of *TOP2A *has been associated with poor cancer-specific survival in MPNSTs [11]. Figure 4b shows the relative expression level of *TOP2A*, *ETV4*, *HOXB7 *and *BIRC5*. Of these four genes, only *BIRC5 *is located within the minimal recurrent region of gain. *TOP2A*, *ETV4 *and *BIRC5 *showed increased expression in all MPNST samples compared to the benign tumors, in most cases more than 20-fold, whereas *HOXB7 *showed approximately similar expression levels in the MPNSTs and the benign tumors. *ETV4 *showed the highest level of expression; its expression was more than 140-fold higher in MPNST9x and more than 90-fold higher in MPNST1 compared to the benign tumors. No clear differences in the expression levels were seen between MPNSTs from patients with poor outcome and MPNSTs from patients who showed disease-free survival (MPNST1 and -13).

The expression levels of miRNAs located within 17q were in addition investigated, in order to see if their expression was increased due to the genomic gain. At the time the analyses were performed, five miRNAs were identified within the minimal recurrent region of gain according to Ensembl; hsa-miR-142p-3p, -142-5p, -301, -21 and -338. The expression level of these miRNAs was determined using quantitative real-time RT-PCR in six of the samples. Figure 4c shows the relative expression level of hsa-miR-142p-3p, -142-5p, -301, -21 and -338. The two miRNAs hsa-miR-142-3p and -142-5p, originating from the different arms of the stem-loop precursor, showed a similar expression pattern. In general, none of the miRNAs showed high expression levels in the MPNSTs compared to the benign tumors, and no clear differences in the miRNA expression levels were seen between MPNSTs from patients with poor outcome and MPNSTs from patients who showed disease-free survival (MPNST1 and -13).

## Discussion

We have used array CGH to analyze DNA copy number changes in a small panel of high-grade MPNSTs, in order to identify recurrent copy number alterations at high-resolution and thereby novel candidate oncogenes and/or tumor suppressor genes. After a recent pathological revision of our tumor collection, seven samples were classified as MPNSTs based on the current classification standard and included in the study. Although this is a small number of samples, the analyses revealed results of potential importance for this malignancy.

Considerably more gains than losses were observed in the seven MPNSTs. Only five of the 40 identified minimal recurrent regions of alteration were losses (see Figure 2a and Table 2), similar to previous observations by others [7,10,13]. However, other studies have reported far more frequent losses than gains in MPNSTs as well [4,14]. Differences in the chromosomal regions altered in sporadic versus neurofibromatosis-associated MPNSTs have previously been demonstrated [6,9,10,13], but the only difference observed here was loss of 11q23.2-q23.3. This region was deleted in the three sporadic MPNSTs, but not in the four neurofibromatosis-associated MPNSTs. However, two of the neurofibromatosis-associated MPNSTs showed loss of other parts of chromosome 11, and the two other minimal recurrent regions of loss in chromosome 11, 11p13 and 11q22.3-q23.1, were deleted in both sporadic and neurofibromatosis-associated MPNSTs [see Additional file 2].

It has previously been reported that patients with neurofibromatosis-associated MPNSTs have a worse survival than patients with sporadic MPNSTs [15,16], but this has not been a consistent finding [17,18]. In this panel of tumors, the two longest surviving patients who showed no evidence of the MPNST tumor after 100 and 216 months, respectively, have neurofibromatosis (Table 1).

The most frequent alterations observed in the seven MPNSTs were gains of regions in 1q, 8p, 9q and 17q, all present in five of the seven tumors analyzed. Within 1q, three minimal recurrent regions were identified. 1q24.1-q24.2 (3.7 Mb) and 1q24.3-q25.1 (3.5 Mb) were gained in five of seven samples, whereas 1q21.1 (1.2 Mb) was gained in three samples. Gain of regions in 1q has not frequently been reported in MPNSTs previously, but it is a recurrent finding in soft tissue sarcomas [19,20] and other malignancies. Since gene expression of six of the MPNSTs studied here has previously been analyzed using cDNA microarrays [12], the expression level of genes located within these regions was investigated, but none of the genes present on the cDNA microarray were over-expressed compared to the median for soft tissue sarcomas (log_2 _ratio > 1) in more than one sample. This was also the case for genes located within the minimal recurrent region in 9q34.11-q34.13 (1.0 Mb).

A minimal recurrent region in 8p23.1-p12 (22.9 Mb) was gained in five of the samples. Within this region, six genes showed increased expression in two or more of the samples [12]. *LOXL2 *and *ZNF395 *were over-expressed in three of the samples. The expression level of these two genes was in addition determined using quantitative real-time RT-PCR, and the expression level was compared to two benign tumors (Figure 3). *LOXL2 *showed more than 3-fold increased expression in three MPNSTs, whereas *ZNF395 *showed approximately similar expression levels in the MPNSTs and the benign tumors. Increased expression of *LOXL2*, a member of the lysyl oxidase family, has previously been shown in colon- and esophageal cancer [21], and it has also been associated with breast cancer tumor grade [22]. Thus, *LOXL2 *may be a candidate target for the 8p23.1-p12 gain in MPNSTs.

Scattered high-level amplification (log_2 _ratio > 1) and homozygous deletion (log_2 _ratio < -1) were observed in some of the tumors [see Additional file 2]. Within 9p22.3-p21.2, homozygous deletion of the region harboring the gene *CDKN2A *was observed in two samples, and heterozygous deletion in one sample [see Additional file 2]. Inactivation of this region is a frequent finding in MPNSTs [23,24], as well as other cancer types [25]. The expression level of *CDKN2A *was determined using quantitative real-time RT-PCR (Figure 3), and the three samples with deletion of the region showed no or very low expression compared to the benign tumors, whereas the other three samples showed increased expression. Notably, the expression level of *CDKN2A *was lower in the four patients with poor outcome than the two patients with disease-free survival (Figure 3).

We observed that all five patients with poor outcome showed gain of the distal part of 17q, whereas the two patients with disease-free survival did not (Figure 4a). Several other studies have also reported that gain of 17q is frequent in MPNSTs [6,7,9,10,13,26], and this alteration has been associated with poor outcome [10] and development of metastasis [26]. These CGH studies have reported the region of gain to be from either 17q22 or 17q24 to 17q25/17qtel. The minimal recurrent region identified in this study, using microarrays, covered 17q23.2-q25.3 (qtel). This region was gained in five tumors, and four of these showed also gain of the proximal region (17q21.2-q23.2).

The expression level of genes located within the minimal recurrent region, as well as the proximal region where four of seven samples showed gain, was investigated. Ten genes showed increased expression relative to the median for soft tissue sarcomas in two or more of the six tumors analyzed [12], although it should be noted that the cDNA microarray only contained probes for about half of the genes in the region (including novel and predicted genes). Three genes were over-expressed in three of the tumors; *ETV4*, *HOXB7 *and *BIRC5*, and the expression levels of these genes, as well as *TOP2A*, were in addition determined using quantitative real-time RT-PCR (Figure 4b). Of these four genes, only *BIRC5 *is located within the minimal recurrent region of gain. *TOP2A*, *ETV4 *and *BIRC5 *showed increased expression in all MPNST samples, in most cases more than 20-fold, whereas *HOXB7 *showed approximately similar expression levels in the MPNSTs and the benign tumors. Even though only tumors from patients who died of the disease showed gain of this region, increased expression of these genes was also seen in tumors from the patients who showed no evidence of the disease. Thus, there were no correlations between the expression level of the genes and patient survival.

*BIRC5*, also known as survivin, is an inhibitor of apoptosis that has been shown to be highly expressed in the majority of cancers, including soft tissue sarcomas and osteosarcomas [27,28]. Increased expression of *BIRC5 *has been associated with chemotherapy resistance, enhanced proliferation, increased tumor recurrence and shorter patient survival [27]. *BIRC5 *is located within a 2 Mb region in 17q25 previously shown to be commonly amplified in MPNSTs [26], and increased expression of *BIRC5 *in MPNSTs compared to neurofibromas and benign schwannomas has been reported [29,30]. Hence, there is considerable evidence suggesting that *BIRC5 *may be involved in MPNST tumorigenesis.

Increased expression of *TOP2A *has been previously associated with poor cancer-specific survival in MPNSTs [11], whereas increased expression of *ETV4*, a member of the Ets family of transcription factors, has been associated with shorter patient survival in colorectal cancer [31] and gastric cancer [32]. Although these two genes were not located within the minimal recurrent region of gain, their associations with poor outcome in MPNSTs (for *TOP2A*) and other malignancies suggest that increased expression of these genes may play a role in MPNST tumorigenesis as well.

Recently, it has been shown that expression of miRNAs can be deregulated in cancer, and that miRNAs may act as oncogenes and tumor suppressor genes [33]. miRNAs are frequently found in regions of DNA copy number aberrations, and a general correlation between miRNA copy number and expression level has been reported [34,35]. In order to investigate if miRNAs may be candidate targets for the 17q gain, we analyzed the expression level of five miRNAs present in the minimal recurrent region (Figure 4c). Compared to the benign tumors, none of the miRNAs showed high expression levels, and no clear differences in the miRNA expression levels were seen between MPNSTs from patients with poor outcome and MPNSTs from patients who showed disease-free survival. Thus, no clear correlation could be found between DNA copy number and miRNA expression in 17q in our samples.

## Conclusion

Our study has identified recurrent copy number alterations in MPNSTs at high resolution, and shows the potential of using DNA copy number changes obtained by array CGH to predict the prognosis of MPNST patients. *LOXL2 *was identified as a candidate target gene for the 8p23.1-p12 gain. Although no clear correlations between the expression level and patient outcome were observed here, the genes *TOP2A*, *ETV4 *and *BIRC5 *are interesting candidate targets for the 17q gain associated with poor outcome, but further validation is required on a larger tumor set.

## Methods

### Tumor samples

Six human sarcomas classified as MPNSTs were selected from a tumor collection at the Department of Tumor Biology at the Norwegian Radium Hospital. One additional sample initially diagnosed as malignant fibrous histiocytoma was included in the study after reclassification to MPNST. Two benign tumors, one BS and one NF, were used as a reference for the quantitative real-time RT-PCR analyses. All tumors were revised at the time of the study by the pathologist (B.B.) and diagnosed according to the current World Health Organization classification. The informed consent used and the collection of samples were approved by the ethical committee of Southern Norway.

Clinical samples were collected immediately after surgery, cut into small pieces, frozen in liquid nitrogen and stored at -70°C until use. Some of the samples were grown subcutaneously in immunodeficient mice as xenografts (suffix x). Animal care was in accordance with the institution's guidelines. Clinical data for all samples are given in Table 1.

### Array CGH

The genomic microarray used contained 4,549 BAC and PAC clones representing the human genome at approximately 1 Mb resolution, as well as the minimal tiling-path between 1q12 and the beginning of 1q25. Detailed information on the construction and preparation of the microarray has been previously described [36]. The microarrays were provided by the Norwegian Microarray Consortium.

Array CGH was performed essentially as described previously [36]. In brief, approximately 500 ng of *Dpn*II-digested total genomic DNA was labeled by random priming using BioPrime DNA Labeling System (Invitrogen, California, USA) and Cy3-dCTP (tumor) or Cy5-dCTP (reference) (PerkinElmer, Massachusetts, USA). Labeled tumor and reference DNA were combined together with 135 μg human Cot-1 DNA (Invitrogen). Hybridization was performed using an automated hybridization station, GeneTAC (Genomic Solutions/PerkinElmer), agitating the hybridization solution for 42–46 hours at 37°C. The arrays were scanned using an Agilent G2565BA scanner (Agilent Technologies, California, USA), and the images were segmented using GenePix Pro 6.0 (Axon Laboratories, California, USA). Further data processing, including filtering and normalization, was performed using M-CGH as previously described [36,37].

### Array CGH data analysis

The complete array CGH dataset for the seven MPNSTs can be viewed in the ArrayExpress microarray database (accession number E-MEXP-869). Clones belonging to chromosomes 1–22 with known unique chromosomal location in Ensembl (v33, Sep 2005) were considered for analysis (3,351 clones). Due to experimental variation in normal control experiments, 22 clones (0.7%) were discarded as described previously [36]. In addition, clones with missing values in three or more of the seven samples were discarded, leaving 3,167 clones for analysis. The remaining missing values were imputed via a K-Nearest Neighbor algorithm normalization using "Significance Analysis of Microarrays" [38].

In order to determine copy number changes, CGH-Explorer v. 2.55 was used [39]. ACE was performed using a false discovery rate of 0.0000. Chromosomal segments showing gains or losses in at least three of seven MPNSTs (≥ 43%) were used to identify minimal recurrent regions of alteration.

### Quantitative real-time RT-PCR

Quantitative real-time RT-PCR was performed using TaqMan Gene Expression and MicroRNA Assays (Applied Biosystems, California, USA). The expression level was determined for the genes *LOXL2 *(assay ID Hs00158757_m1), *ZNF395 *(assay ID Hs00608626_m1), *CDKN2A *(assay ID Hs00924091_m1), *TOP2A *(assay ID Hs01032127_g1), *ETV4 *(assay ID Hs00385910_m1), *HOXB7 *(assay ID Hs00270131_m1) and *BIRC5 *(assay ID Hs00153353_m1). The genes beta-2-microglobulin (*B2M*, assay ID Hs99999907_m1), glyceraldehyde-3-phosphate dehydrogenase (*GAPDH*, assay ID Hs99999905_m1) and TATA-box binding protein (*TBP*, assay ID Hs99999910_m1) were used as endogenous controls for normalization of gene expression. The expression level was determined for the miRNAs hsa-miR-142-3p (assay ID 464), hsa-miR-142-5p (assay ID 465), hsa-miR-301 (assay ID 528), hsa-miR-21 (assay ID 397) and hsa-miR-338 (assay ID 548). The small nuclear RNAs RNU6B (assay ID 1093) and RNU24 (assay ID 1001) were used as endogenous controls for normalization of miRNA expression.

Frozen tumor tissue was pulverized in liquid nitrogen, and total RNA was extracted using Trizol (Invitrogen) according to the manufacturer's instructions. The total RNA was further purified using the RNeasy Mini Kit (QIAGEN, California, USA) as described by the manufacturer, with a few modifications in order to preserve the miRNAs. In brief, after addition of buffer RLT and vortexing, 3.5 volumes of 100% ethanol was added and mixed by vortexing. The sample was subsequently applied to the RNeasy Mini column. Washing with buffer RW1 was not performed. Universal Human Reference RNA (Stratagene, California, USA) was used as a reference for the gene expression assays. cDNA synthesis and real-time PCR were performed essentially as described in the protocols supplied by the manufacturer (Applied Biosystems). The PCR amplification was performed using the ABI 7500 Real Time PCR System (Applied Biosystems).

Each gene expression assay included (in duplicate) a standard curve of four serial dilutions of the Universal Human Reference RNA cDNA (ranging from 50 ng to 50 pg), 10 ng of each tumor cDNA and a no-template control. The expression levels were determined from the standard curves as described by the manufacturer. The expression level of each gene was normalized with the average expression of the three endogenous controls. The expression level of each gene in the MPNSTs was determined relative to the average expression of the benign tumors.

Each miRNA expression assay included (in duplicate) 0.3 ng of each tumor cDNA and a no-template control. The experiments were done twice, and the average values were used. The expression levels were determined using the comparative C_T _method as described by the manufacturer, and the expression level of the miRNAs was normalized with the average expression of the two endogenous controls. The expression level of each miRNA in the MPNSTs was determined relative to the average expression of the benign tumors.

## Competing interests

The authors declare that they have no competing interests.

## Authors' contributions

SHK performed the array CGH experiments, all analyses and drafted the manuscript, MS performed the quantitative real-time RT-PCR experiments, HOO collected the clinical data, HMN performed the microarray expression profiling, BB revised pathologically all the clinical samples, OM and LAMZ conceived of the study, participated in its design and coordination and helped to draft the manuscript. All authors read and approved the final manuscript.

## Supplementary Material

Additional file 1 Whole genome DNA copy number profile for seven MPNSTs. Click here for file

Additional file 2 Identification of minimal recurrent regions in MPNSTs using ACE. Click here for file
