# Supplementary material for: DNA copy number changes in high-grade malignant peripheral nerve sheath tumors by array CGH
Source: Mol Cancer. 2008 Jun 3;7:48. doi: 10.1186/1476-4598-7-48 (PMC2442610; doi:10.1186/1476-4598-7-48)
Supplement: Additional file 1 — Whole genome DNA copy number profile for seven MPNSTs. [file 1476-4598-7-48-S1.pdf]

MPNST1

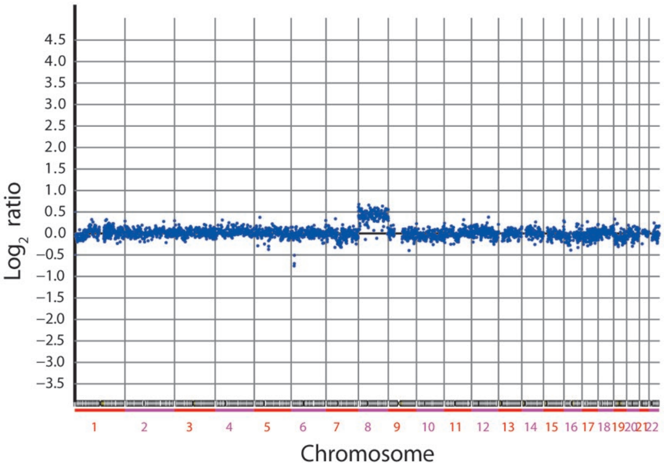


MPNST2

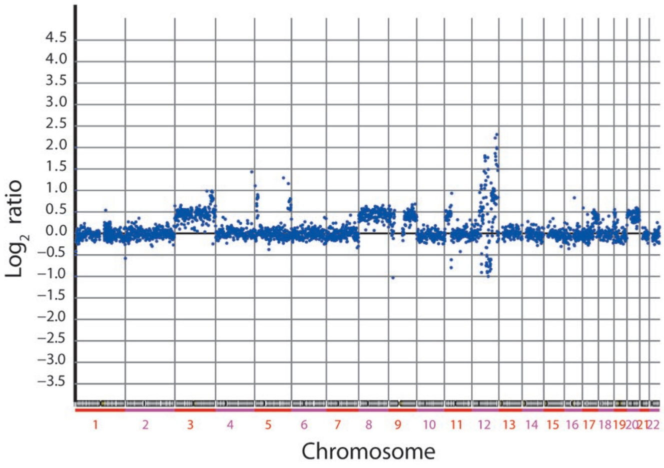


MPNST5

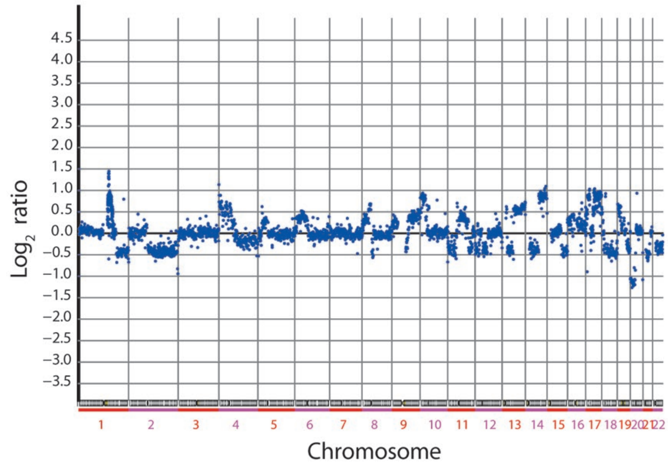


MPNST7

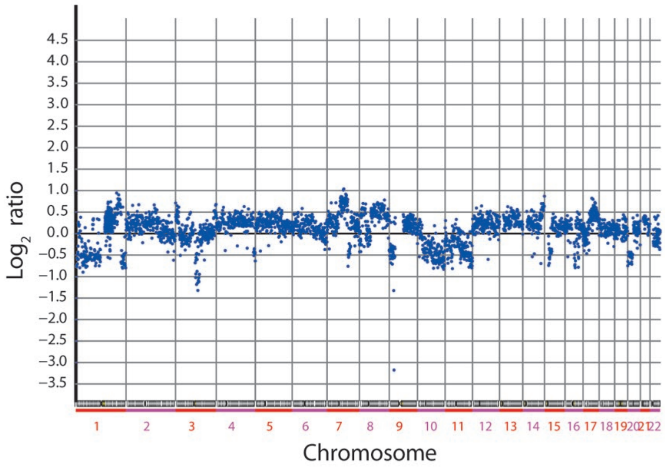


MPNST8x

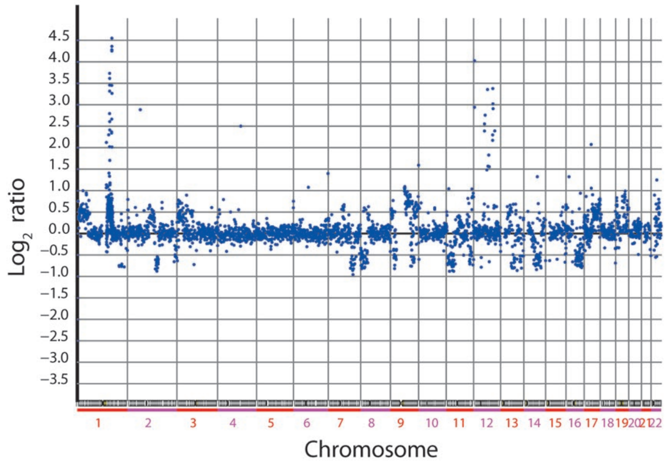


MPNST9x

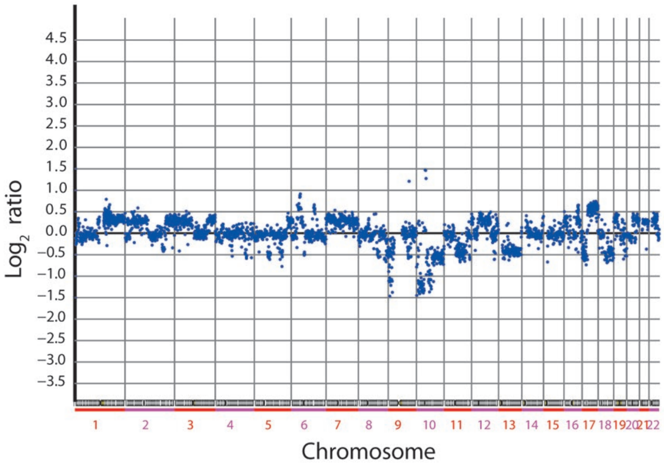


MPNST13

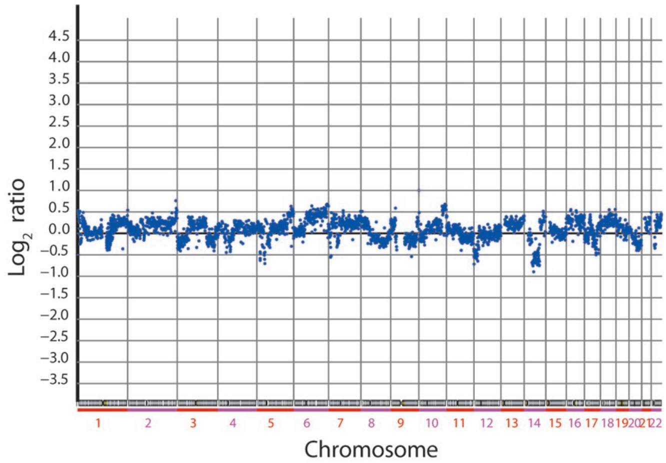


**Supplementary Figure 1.**
Whole genome DNA copy number profile for seven MPNSTs. $Log_2$ ratio for each of the genomic clones is plotted according to chromosome position.

Kresse *et al.*
